# Consumer Health-Related Activities on Social Media: Exploratory Study

**DOI:** 10.2196/jmir.7656

**Published:** 2017-10-13

**Authors:** Arcelio Benetoli, Timothy F Chen, Parisa Aslani

**Affiliations:** ^1^ Faculty of Pharmacy The University of Sydney Sydney Australia; ^2^ Department of Pharmaceutical Sciences State University of Ponta Grossa Ponta Grossa Brazil

**Keywords:** social media, social networking sites, Facebook, YouTube, Wikipedia, Twitter, health, consumers, patients, focus groups

## Abstract

**Background:**

Although a number of studies have investigated how consumers use social media for health-related purposes, there is a paucity of studies in the Australian context.

**Objective:**

This study aimed to explore how Australian consumers used social media for health-related purposes, specifically how they identified social media platforms, which were used, and which health-related activities commonly took place.

**Methods:**

A total of 5 focus groups (n=36 participants), each lasting 60 to 90 minutes, were conducted in the Sydney metropolitan area. The group discussions were audiorecorded and transcribed verbatim. The transcripts were coded line-by-line and thematically analyzed.

**Results:**

Participants used general search engines to locate health-related social media platforms. They accessed a wide range of social media on a daily basis, using several electronic devices (in particular, mobile phones). Although privacy was a concern, it did not prevent consumers from fully engaging in social media for health-related purposes. Blogs were used to learn from other people’s experiences with the same condition. Facebook allowed consumers to follow health-related pages and to participate in disease-specific group discussions. Wikipedia was used for factual information about diseases and treatments. YouTube was accessed to learn about medical procedures such as surgery. No participant reported editing or contributing to Wikipedia or posting YouTube videos related to health topics. Twitter was rarely used for health-related purposes. Social media allowed consumers to obtain and provide disease and treatment-related information and social and emotional support for those living with the same condition. Most considered their participation as observational, but some also contributed (eg, responded to people’s questions).

**Conclusions:**

Participants used a wide range of social media for health-related purposes. Medical information exchange (eg, disease and treatment) and social and emotional support were the cornerstones of their online activities. Social media appears to be used as a key tool to support disease self-management.

## Introduction

Since the mid-1990s, the Internet has become widely available enabling consumers (including patients) to freely search for health-related information. This increased access has changed the role of consumers from passive recipients to active consumers of health information [1]. Initially consumers obtained health information from the Web on a noninteractive, unidirectional platform. However, with the advent of social media, consumers not only access but also create and share online content. As a result, it has been claimed that social media has supported a shift from the informed patient to the participative patient [2]. Social media can be defined as Web-based technologies and applications whose content is created by the users [3]. It can be divided into content sharing platforms (eg, blogs, YouTube, Wikipedia) and relationship building platforms (eg, Facebook, LinkedIn) [4]. The latter primarily consist of social networking sites, social media platforms in which users create a profile and establish connections with other users within it, creating a network [5].

The interactive and participatory nature of social media has afforded consumers not only a greater opportunity to access health-related information but also a venue to provide health-related content to others [6]. Although online communities existed before the advent of social media, the appearance of social networking sites fostered its growth, and they have become very popular [7]. Disease symptoms, complications and prognosis, examinations, and treatments are popular topics discussed in these online communities [8]. One very popular social media platform widely used for health-related purposes is Facebook, where a wide array of health topics, pages, and groups is present [9]. Studies have shown that interactions among peers in these online groups have been beneficial for those living with chronic conditions such as diabetes [10,11], epilepsy [12], and breast cancer [13,14]. In these online communities people can provide and receive social support, cultivate companionship, exert social influence, and communicate with one another [15,16]. As chronic conditions are currently the leading cause of morbidity and mortality [17,18], it is important to fully comprehend how social media is being used for health-related purposes in order to optimize its use and implement new ways of supporting its application for health promotion. As long-term use of medications is a key component of chronic disease management, it is also imperative to investigate how online interactions between peers influence medication-related decisions.

Previous research has provided a limited understanding of the different social media platforms consumers use for health-related purposes, particularly in the Australian context. Understanding current online health practices of consumers is important for the development of online health resources and services. This research was designed to investigate consumer self-reported participation in social media and other online forums, with the specific objectives of (1) investigating how consumers identify social media platforms for health-related purposes, (2) exploring the social media platforms used, and (3) examining the health-related activities that commonly take place.

## Methods

### Focus Groups

A qualitative study was undertaken using semistructured focus groups to explore consumer experiences, opinions, and perceptions about their use of social media for health-related purposes. Specifically, the group discussions investigated how consumers identified and chose social media, what platforms they used, and what kind of information they were looking for and providing to others. A qualitative approach was chosen because it can expose subtleties and complexities about the topic under investigation that are often missed by more positivistic inquiries [19]. Focus groups are more advantageous than surveys because participants do not have to write their answers down, which can be time consuming, and therefore they may provide more information with more explanations and detail. The facilitator can gain further information from them, participants can seek clarification if they do not understand a question (which they cannot do when completing a self-reported survey), the facilitator can ask follow-up questions and seek clarification from participants based on the responses provided, and other participants present in the focus group providing their responses can trigger participants’ memory and therefore aid in obtaining more complete data. The focus groups were semistructured as the discussions were guided by an interview protocol (Multimedia Appendix 1) containing key, broad, open-ended questions allowing participants to elaborate on their responses. Focus groups are also advantageous compared to other qualitative methods. They are an efficient way of gathering the views of several individuals simultaneously [20] and uncovering important constructs that may not be tapped through individual interviews [21]. Focus groups are highly recommended for health services research [22]. In the field of pharmacy, they have been used to explore important areas of research such as consumers behaviors [23]. The literature also recommends further investigation about consumer online communication and participation in forums using qualitative methods such as focus groups and in-depth interviews [24]. Therefore, focus groups were selected as the most appropriate research method. This study received approval from the University of Sydney Human Research Ethics Committee prior to its commencement.

### Participants and Recruitment

#### Inclusion Criteria

Participants in this study consisted of adults aged 18 years and older with chronic conditions (ongoing health problems that have occurred for 3 months or longer) managed by medications [25]. Even though certain chronic conditions can be managed without medication, this study required participants to be on at least 1 chronic condition medication so that it would be possible to explore how they searched for, discussed, and provided information about their medications and medical conditions on social media. Other study inclusion criteria were that participants had used social media to discuss health-related issues in the last 12 months and were able to participate in the study without the assistance of a translator.

#### Recruitment

A recruitment agency identified participants meeting the inclusion criteria from the metropolitan Sydney area. All participants received detailed information about the study background, aims, and researchers conducting the study. Out of 40 participants recruited, 36 took part in this study. Each participant was reimbursed Aus $80 (US $62) for their time and travel expenses. All participants received verbal and written information about their participation and a consent form.

### Data Collection

The focus groups (n=5) were conducted in 3 distinct geographical areas of Sydney to capture consumers from a range of socioeconomic backgrounds. The focus groups were held in venues commonly used for meetings and group discussions. Upon arrival at the focus group venue, participants were provided with a participant information statement and asked to complete a consent form and demographic questionnaire. Discussions lasted between 60 and 90 minutes and were facilitated by PA, a female pharmacist and academic experienced in conducting focus groups. Discussions were audiorecorded with permission from all participants. Notes were taken in order to facilitate data analysis. Focus groups were conducted until data saturation [26] was observed, which was at the conclusion of the fourth focus group. One extra focus group was conducted for validation purposes.

### Data Analysis

The analytical process started during and in parallel with data collection [27]. Note-taking during the focus groups and debriefing after each session ensured that important information was not missed and constituted a preliminary analysis [21]. All discussions were audiotaped and transcribed verbatim with tracking of individual speakers, without identifying the individual. The qualitative data from the focus groups were analyzed using the inductive approach of thematic analysis [28] to derive themes and subthemes. Themes are “best used to describe an integrating, relational statement derived from the data that identifies both content and meaning” [29]. Additionally, some of the findings were descriptively analyzed as described by Sandelowski [30], as these findings were best suited to descriptive rather than thematic analysis (eg, what social media platforms were used; where, when, and how frequently social media was accessed). Therefore, a blended approach combining descriptive and thematic analysis was employed to interpret the focus group discussions. This combination allowed for the description of findings that did not lend themselves to being thematically analyzed and for the derivation of themes and subthemes through the iterative process of comparing and contrasting the codes within and between the focus groups. All discussions were coded by AB, and the coding process, including its classification into themes and subthemes, was discussed with PA. Repeated reading of notes and transcriptions was the first analytical step in order to gain familiarity with the data and knowledge of the content in each group. Next, the transcriptions were coded line-by-line with the assistance of NVivo 11 (QSR International) computer software. The coding process was open and not restricted by theoretical framework. It was dynamic and iteratively evolving throughout the analysis. An inductive approach [31] assured a data-driven process. Codes with a repeated pattern across the data (ie, codes with similar or nearly similar meanings) were collated and grouped into subthemes and later assembled into overarching themes. Themes were carefully named according to their overall content.

## Results

### Participant Characteristics and Major Themes

A total of 36 participants took part in 5 focus groups (Multimedia Appendix 2). Overall, there were slightly more men (19/36, 53%), with the majority of participants having been born in Australia (26/36, 72%). Participants had a range of different chronic disease states including hypertension, depression, anxiety, cancer, arthritis, and Crohn disease.

Thematic analysis of the focus group discussions identified 7 key themes related to the objectives. The emerging themes and their subthemes are presented in this section together with illustrative quotes. Table 1 provides a summary of the themes derived from the data organized according to the respective study objectives.

**Table 1:** Themes derived from the data.

Study objectives	Themes	Major qualitative analysis employed
Investigate how consumers identify social media platforms for health-related purposes	Search facilitates and precedes access to social media platforms	Descriptive
	Social media has ill-defined boundaries	Thematic
Explore the social media platforms consumers used	Social media platforms used for health-related purposes	Descriptive
	Access to social media platforms	Descriptive
Explore the health-related activities that commonly take place	Health-related activities that take place on social media Getting more information Fulfilling a social need	Thematic
	Observing versus posting	Descriptive
	Social media identity and privacy	Thematic

### Search Facilitates and Precedes Access to Social Media Platforms

Overall, 2 approaches were adopted for accessing social media for health-related purposes. The most popular method was to use a general search engine such as Google. As consumers were using search engines to search for health information (eg, condition and treatment), they ended up finding online platforms where they could not only learn from peer experiences but also interact with other consumers. The top hits on the first page were generally the most frequently accessed ones. Subsequent pages would be accessed only when nothing of interest could be found in the first one. A shortcut to finding online health forums was to insert the word “forum” along with other key terms, such as disease name.

The second search approach was to use the social media platform itself. For example, in order to find disease-specific groups or related pages within Facebook, participants would use the Facebook search engine feature and type in the disease for which they were searching. Regardless of which strategy was used, most participants resorted to a general search engine for further research on the topic of interest.

### Social Media Has Ill-Defined Boundaries

This research did not aim to systematically assess participant understanding of social media. However, it was observed that in some cases, the concept had no clear boundaries with other online platforms. When asked about their use of social media, several participants mentioned websites not technically classified as social media, such as search engines, Internet browsers, and health websites. For example, websites with some sort of user engagement or with a chat room were commonly mentioned: Medscape, Blue Board, Psych Central, WebMD, Mayo Clinic, and Beyondblue. Chat rooms, in particular, were used by several participants as a venue for interacting with peers dealing with the same condition. Participants were free to express and discuss their online behavior. However, when a misunderstanding about social media platforms was noticed or they spoke about nonsocial media platforms (eg, “dot.com” websites), they were refocused back to social media. Importantly, a user-friendly definition of social media with examples (eg, Facebook, YouTube, Wikipedia, blogs) was provided at the beginning of the discussion to ensure clarity in the discussion topic.

### Social Media Platforms Used for Health-Related Purposes

Participants used a range of social media platforms for health-related purposes including Facebook, Wikipedia, YouTube, blogs, and Twitter.

Most participants were active on Facebook, and only a few did not have a Facebook profile. Some were members of disease-specific Facebook groups, such as an arthritis group, as their approach to using Facebook for health-related information. Some of these disease-specific groups had an international membership. The use of Facebook for health-related purposes was regarded as very convenient since such use was integrated into the general use of the platform.

...the good thing about Facebook is that it’s not just about your health issues. It’s about the whole world and all the groups that you're on. So you don’t have to sort of...you can just flick through it in the morning and cover everything.

FG1, m4

Belonging to a Facebook group was very practical as participants did not need to leave it to access health-related content by browsing different websites or platforms. The group activities appeared on their Facebook newsfeed.

Wikipedia was a source of health information frequently accessed by most participants. It also served as a way of reaching other sources of health information through its references and external links. Despite its common use, some participants expressed mistrust in the content found on the Wikipedia.

I always trusted it and then, I looked up something I knew the answer to, and it was wrong. And I thought ‘this is not good’...so, yeah, I’ve now taken a more...I’m not as wide-eyed when it comes to Wikipedia.

FG2, f15

As a consequence of not fully trusting Wikipedia, some consumers developed double-checking mechanisms for the information retrieved (ie, crosschecking the information found on Wikipedia with another online source.) Participants reported that they had not updated any Wikipedia content.

The majority of participants accessed video-sharing platforms, but very few used them for health-related reasons. YouTube, the only video-sharing platform cited, allows participants to access health information and peer experiences in a video format. The only use of YouTube for health-related reasons reported was to learn about medical procedures and to watch surgeries participants had undergone or were to undergo.

I wanted to know how that process was done, the ultrasound...and injecting, cortisone injection. So I wanted to know what the procedure was. So, I went through the whole thing.

FG3, f17

While the graphic details of health procedures in video footage were not attractive to some participants, the discussion sparked interest in accessing video footage for health-related purposes among those who had not used YouTube for that purpose to date. No participant had uploaded videos about their own health experiences.

*I use YouTube but I never thought about* [it] *for health...but probably there must be something. I’m curious to see.*

FG3, f18

Blogs were considered good platforms to learn about other people’s health experiences. Most participants would read blogs but not write on them. Only 1 participant was blogging about his own experiences with the disease and therapeutic breakthroughs in the area.

I like to blog my own experience so that others can relate and get the benefit. And if I find something which is innovative, then that’s something I would like to share. Because I tend to read a lot of medical journals, the original research findings.

FG1, m6

However, blog use was much less common among participants. Blogs were surpassed by newer platforms like social networking sites.

...since Facebook came along, blogs went out the window for me.

FG2, m13

A few participants reported using Twitter, mostly to access general information or news. Only 1 participant used Twitter to obtain medical information.

...talking about drugs and their effects. Legal drugs... Nobody follows me. But I follow them. I don’t post anything but I read all that stuff.

FG1, m1

### Access to Social Media Platforms

This theme describes how long participants had been accessing social media platforms, how often, how they were accessed, and when social media was used. The duration of time consumers had been using social media varied. While the majority reported having using it for a long time, some had started using it later, with a few participants reporting that they had only recently started using social media.

The frequency of social media use among participants ranged from “virtually on it all the time” to “a few times a week” to “daily” to “whenever you’ve got a pocket of time.” The frequency of social media use was related to its availability on several electronic devices, such as computers, laptops, tablets, and mobile phones. The devices used for accessing online platforms varied depending on where participants were at the time of access (eg, traveling, at home, or at work). For example, portable devices, particularly hand-held ones like mobile phones, were commonly used for social media access during commuting time on public transportation, while laptops and tablets were mainly used at home, and desktops were mostly used at work.

For those working in offices, social media was constantly present on their computer screens. Facebook, for example, was accessed multiple times a day by those working in front of a computer. However, not everyone was comfortable using social media at work due to job restrictions or privacy concerns; some preferred to only access social media during their free evening time at home.

Some participants preferred to regularly access social media for health-related activities in the evenings at home, with one participant recognizing that it would be wise to allocate time to conduct online health-related activities.

I should be doing it when I’m on an even keel or probably allocating a certain amount of time to do proper research and understand a little bit more about the types and nature of the medications.

FG, m13

Most commonly, a new health problem or a disease flare-up were triggers for online engagement.

I’ve got Crohn’s disease and it comes and goes. So, I could go for 6 months, I’m fine, I don’t need to get any help. But, if I’m going through a bad period, and I’m finding it really hard going then I’ll go on to forums, just look up anything that I can find, just to get me through.

FG5, f34

Indeed, it was expressed by a few participants that social media for health-related purposes was only used when needed.

I only go on it when I’m not well.

FG5, f34

I don’t kind of scroll through this all the time, I only use it when I need it.

FG5, m35

### Health-Related Activities That Take Place on Social Media

#### Reasons For Social Media Engagement

Several reasons were mentioned by participants for engaging in social media platforms for health-related reasons. Information (ie, accessing user-friendly health information, especially other people’s experiences and treatment information) and social support (ie, relating to people with the same problem and providing and gaining encouragement) were the main motives for accessing social media for health-related purposes.

#### Getting More Information

Obtaining user-friendly health information was one of the major reasons for using social media for health-related purposes. Social media also had the advantage of being interactive, with participants being able to ask questions and provide answers and comments.

Participants were interested to know what other people with the same condition were being treated with and to learn about other people’s experiences with the same medication.

I’m on medication of course I did some research on medication. And also, I just want to know what other people take and what they eat and what they do.

FG2, f12

I look for a testimonial, the history of using it [medication], the experiences they’ve had, the side effects, and so forth. Whether it was effective.

FG2, m11

Sometimes this represented a double-checking mechanism in order to verify if the medication prescribed for them really was the adequate course of action to be taken.

Side effects were a major trigger for online research, particularly when starting a new medication. Participants stated that the information presented on pharmaceutical company websites did not meet consumer needs since they provided too much general information, particularly for side effects. Therefore consumers preferred to hear what was really happening with people taking the medication.

*...if it* [side effect] *really happens to people. So I think it’s better to talk to somebody who is really using medication.*

FG3, f18

Interacting on social media with peers influenced the way most participants perceived their treatment, which could in turn impact medication adherence.

...my wife says ‘those new tablets the doctor gave me is giving me pains in the chest’...and I’ll go ‘let’s have a look at that’...and all of a sudden there's a forum and ‘don’t take them.’

FG2, m13

Social media was also used to identify and learn about complementary and alternative medicines, especially as participants felt that doctors were reluctant to provide such options.

*I go in and ask people ‘what are you taking?’ So it’s not chemicals, not prescription drugs. And I’ve been suffering from GORD for years. And people start taking apple cider vinegar. So, every day I drink apple cider vinegar and I’m throwing the* [medicine name] *tablets away...with my wife’s medication as well, I look to see if there's alternative medicines for her as well so we can start getting off prescription drugs.*

FG2, m13

Provision of incomplete health information from health care professionals was another reason for resorting to online sources

...there is a forum that I basically sometimes belong to on Facebook, for one condition, my arthritis...so, when I’m considering a medication, when I’m concerned about a contradiction, because I find my doctor, despite his best efforts, is not very thorough. Same with the pharmacist.

FG2, m11

#### Fulfilling a Social Need

An important activity reported was to gain social and emotional support from others with the same problem, particularly after receiving a diagnosis of a chronic condition, to feel that “this is not the end of the world” (FG1, f5).

It was emphasized that getting support from people going through the same health problems was really important because people could easily relate to one another. It was mentioned that although people receive help and support from family and friends, the fact that they were not experiencing that same problem themselves prevented them from completely understanding what the disease bearer was going through or experiencing.

Additionally, consumers were resorting to online support because they could not find the support they needed from their regular health services. This revealed a problem within the way the current health care system: a lack of a holistic approach to address consumer needs. It was revealed that participant needs were not only medical and therapeutic but also social.

...you find like-minded people, people you haven’t met before but like posting things that are really helpful. And you feel like you can find supports there. And you can go into support groups. And like, actually meet up and stuff like that. So, I think that’s really a great part of today’s world. Like, I find it very hard to go to a support group within a hospital that a hospital organizes, but you can find...

FG5, f32

### Observing Versus Posting

The majority of participant engagement in social media was observational (ie, accessing and reading health information rather than providing).

I don’t usually post. I usually just go in to read other people’s, to get experiences and see if I can learn something else about what's out there. If I’m trying out a new pump device, I’ll try and get people’s feedback about what their experiences with health insurance or with the pump itself have been.

FG2, f10

Participants refrained from contributing if they felt that they would not add new or relevant information.

*I don’t tend to give because I find a lot of it is already there. So, like, yeah, I’ll just be repeating what other people* [say]...

FG3, m19

A participant even expressed a feeling of guilt for not being an active contributor on online forums, especially as he gained information from them. Another participant raised the legal responsibility for medical advice on health-related social media groups as a reason for not providing information.

...because someone could say, I took his advice and now look at what it’s done to me. It’s made me so sick I want to sue them. I want to sue him for telling me the information.

FG2, m9

Nevertheless there were some participants who were very active in providing information, and in general, participants seemed to be willing to contribute as long as they considered themselves knowledgeable about the topic (disease condition or treatment). Those who were actively contributing were comfortable because most of the time the information shared was related to their own experiences.

I’m very comfortable because I’m not really giving out information. I’m just sharing what my experiences are. So I’m not really advising somebody this is what happens. I just say, well, this happened to me and this works for me, those sorts of things.

FG1, m7

Some participants expressed their approximate involvement: “80% absorbing, reading and then 20 basically would be contributing” (FG2, m11); “mine is about 70-30. I observe about 70 and post about 30” (FG4, m26); “but yeah probably 95 to 5” (FG4, f28); “I’m 95-5” (FG4, m23). However active contribution could increase if more people were attracted to the discussion.

I’m probably about 60-40...absorbing 60, contributing 40. But once I get going, and then all of a sudden, bang someone’s asking me a question back. I’m like ‘hang on, I’m out here alone, people are reading what I’m typing’...so then I’m back again and then 2 people come back and then all of a sudden it’s good conversation—we’ve got 100 people in the conversation.

FG2, m13

A genuine desire to help others going through the same health issues was a driver for being an active contributor on online forums.

I feel it is essential as well...because the interested parties can benefit. Even if one person gets some additional benefit due to your experience, it’s well worth your time.

FG1, m6

### Social Media Identity and Privacy

Several approaches dealing with social media identity and privacy were identified. They ranged from total openness, such as consumers using their own names and pictures on social media profiles and online forums, to participants restricting the availability of their private details. A few participants were totally open about their identity while interacting on social media and did not consider it problematic to have friends and contacts seeing their online health-related activities.

I’m not concerned about people seeing a perception of my identity just because I’ve contributed to a discussion forum...and I’m really aware of the fact that if I am discussing something in a public forum, it’s something that I’m quite happy for everyone to know.

FG2, m11

Some were using their first name only, instead of their full name, to avoid complete disclosure of their personal information. Some preferred to omit certain personal details, such as surname or date of birth.

Another approach was the use of social media privacy settings. Participants felt that this safeguarded their privacy and confidentiality of the health information posted online. In order for this process to be effective, the social media friends or contacts had to be organized into different lists according to their interests (ie, health-related contacts). Then future posts could be seen only by those in selected lists chosen as the audience.

...that’s why I have groups. I have different subgroups. Like then you can check like I’m sharing this and I want only these people to know, or I’m sharing that and anybody to know it.

FG1, f8

The use of privacy settings, however, was not perceived to be a complete guarantee of privacy for 2 reasons: social media platforms were perceived to benefit from providing participant details to third parties for a profit and social media platform privacy policies could change unexpectedly.

Those who were more concerned about online privacy resorted to the use of pseudonyms or avoided using their own pictures in social media profiles. It is noteworthy that anonymity on social media platforms evoked different opinions. On the one hand, it was mentioned that anonymity could have a deleterious effect on the trustworthiness of the information provided online. On the other hand, the use of an alias was regarded as crucial by some participants to not only safeguard their privacy but also to foster the provision and sharing of personal information needed for peer discussions. The supporters of anonymity emphasized that the content of the information and the way it was presented was more important than the source’s identity.

The reasons provided for keeping online activities private ranged from personal concerns (ie, not wanting friends to know their health problems) to stigma associated with certain diseases and the risk of impacting their professional life (eg, worried about losing their jobs because of their medical condition).

## Discussion

### Principal Findings

This study sheds light on how consumers with chronic medical conditions and on medications were accessing social media for health-related purposes, which social media platforms they were accessing, and how they used social media for health-related purposes. Although focused on the Australian context, these findings may be transferable to other similar settings due to the ubiquitous nature of social media and the ability to access social media from any part of the globe with Internet access.

Despite the reported widespread use of social media, it was interesting to note that general search engines remained the key starting point for online health searches. This finding confirms and helps to consolidate the prominent position of general search engines as the initial and most used strategy to locate online health information [32-37]. This study further highlights that even though participants were seeking social media platforms for health-related information and peer interactions, very few knew how to effectively search within social media platforms themselves. This may be due to the limited understanding of search engines and how to effectively and efficiently search in the online environment. However, once certain social media platforms were used for health-related reasons, consumers tended to keep using them and use the search function within social media platforms to locate topics of interest. As more and more health-related services and other daily services are moving to an online interface, it is essential that consumers are better educated on how to efficiently search, access, and effectively use social media platforms as well as other platforms. It is possible that their limited searching abilities narrow the social networking sites and other online sites they can access for information.

One important factor leading consumers to use social media for health-related purposes was the fact that these platforms were already part of their online daily activities, and incorporating a health component was easy and very convenient. The high availability of mobile phones has afforded easy access and therefore, participants do not need to be at home or in the office and can conduct searches and interact online even while in transit. This important finding demonstrates that consumer access and experience with social media platforms for nonhealth-related reasons has been easily extended and applied to health-related purposes and health-specific social media platforms. More and more consumers are therefore online, with the ability to access people online becoming today’s norm. This increased access to consumers provides a significant opportunity for health care professionals and the health care system that should be capitalized for better patient health outcomes.

The range of social media platforms used highlights the diversity of consumer health-related needs and the importance of having a range of sources of information that can be accessed and interacted with online. Not surprisingly, Wikipedia was commonly used to access health-related information. Besides being user-friendly, Wikipedia appears on the first page of most searches and is considered a prominent source of online health information [38]. The common use of Wikipedia for health information reported in this study strengthens the call for the medical community to join in editing Wikipedia entries in order to ensure their accuracy and also to use Wikipedia as a tool for global public health promotion [39]. The study findings demonstrated that some consumers do contribute to social media (in particular via social networking sitess, such as Facebook) by providing information, responding to other consumers, and supporting people. Therefore, Wikipedia, as a commonly accessed social media, could provide a novel opportunity for consumers to contribute; for example, health-related entries could have a section about consumer experiences and testimonials where first-hand information about disease and treatment could be provided by people. Additionally, links to other platforms providing experiential knowledge (such as YouTube clips) could be included in this section. As demonstrated in this study, consumers value each other’s knowledge and experiences, and entries in Wikipedia are likely to be welcomed by consumers.

This study has shown that consumers were actively searching for health information online and interacting with peers for health-related reasons. Despite having access to health care professionals, consumers felt they were not getting as much information as they felt that they needed. This is in line with previous research that has identified lack of information provided by health care professionals as a reason for consumers resorting to online information [35,40,41]. However, this study has also demonstrated that support is a key reason for consumers going online. This is an important finding and highlights the gap in the current health care and social support patients with chronic medical conditions are receiving. The participants in this study have voiced this gap in their overall health care which they felt they were addressing through engagement with social media. Participation in online groups creates a sense of belonging to a community and being connected to others [42]. This online connection with peers provides an avenue to vent emotional difficulties [43-46] and obtain emotional relief from peers [44,45]. This emotional support could positively impact consumer ability to cope with problems [44,46-48] and increase self-esteem and confidence [49]. Additionally, social and emotional support obtained online from peers can improve disease self-management and control [42,50].

Most of the study participants had a passive role in the social media groups and online communities they belonged to (ie, they were reading much more than they were contributing). However, they appeared satisfied with this level of active engagement, although there were comments that they contribute when they felt that they had something new and valuable to add. This low level of active contribution is in agreement with findings from a US survey that revealed that less than 15% of Internet users were engaged in the creation of online content [51]. Even smaller numbers were reported in a UK study, which found that only 7.5% of users were responsible for posting most of the health-related content [52]. It has been claimed that writing about health problems could have a positive effect on reducing emotional distress [53]. In contrast, another research study concluded that observational participants (also called lurkers) benefited as much as those who actively contributed [45,50]. This is certainly a topic that needs further exploration because if providing information really has a positive impact, social media has a great potential to improve the well-being of consumers as identified in this study.

One explanation for the low contribution found in this study could be the perceived requirement to disclose a great deal of personal information on social media [54]. This study has shown that consumers were concerned about their privacy when using social media for health-related purposes, with some participants concealing their identities to remain anonymous when discussing and sharing health information online and others using pseudonyms. These strategies were adopted by the participants so they could still participate and gain benefits from social media engagement. Consumers could be more active since anonymity afforded them the opportunity to express themselves truly and freely [55,56]. Consumers therefore value social media (the information they can share and the social support they can provide and receive) enough to take extra steps to ensure their privacy and still engage in social media. Moreover, the online anonymity is regarded as an important facilitator for full participation of patients suffering from conditions with high levels of stigma, as they can participate without fear of judgment [57]. It is known that social networking sites have created the privacy paradox, as users disclose personal information on social media and at the same time are concerned about their privacy [54]. This privacy paradox can impact the extent of information shared on social media. Facebook has afforded its users the ability to control their profile and activity visibility through the use of privacy settings, which were used by some of our participants. But as personal information disclosure is the default, users have to be vigilant and make an effort to control what is public or private, and of course they have to first be aware of the privacy settings and how to use them. The use of privacy settings, however, is an issue for all users of social media. A 2012 survey showed that almost 60% of general social media users set profile privacy, despite half of them reporting some difficulty in the task [58].

### Limitations

The findings of this study should be considered in light of certain limitations. First, the findings despite providing useful insights are not intended to be generalizable due to the qualitative approach used. Additionally, all participants were recruited from the Sydney metropolitan area, which might restrict the transferability of findings to other populations (ie, it may not be representative of a cross-section of Australian consumer experience and views related to the use of social media for health). Second, it was not possible to completely isolate consumer activities on social media platforms from other online platforms, such as websites. It seems that consumers and regular users of social media do not see a clear and definite separation between dot.com websites and social media platforms and instead see an evolution within the same concept. The facilitator ensured that the focus was always on social media platforms. Third, the focus group approach, despite serving as a way of stimulating participants to express their experiences and opinions, could also have deterred some participants from fully expressing themselves. To minimize this, all focus groups were run by an experienced facilitator who ensured that each participant could report on their experiences and opinions. Last, due to the dynamic nature of social media, the findings represent the situation at the time of the study only and patterns of use might change quickly.

### Conclusion

Consumers used a wide range of social media platforms for health-related purposes, accessing social media at home, in transit, and in the workplace. Several electronic devices, in particular mobile phones, were used to access social media. Consumers still relied heavily on search engines for their initial health searches, but explorations within social media platforms were also mentioned. Participants observed far more than they actively posted on social media. They wanted to learn about their disease and treatment (including potential side effects) and to provide and seek social and emotional support. Identity and privacy was a concern but did not prevent consumers from fully engaging with a community of peers dealing with the same health problem. Social media platforms provide important opportunities for health care professional involvement in patient care, from public health initiatives to treatment and monitoring of patients.

## Appendix

Multimedia Appendix 1 Focus group discussion guide.

Multimedia Appendix 2 Participant demographics.
