# Supplementary material for: Consumer Health-Related Activities on Social Media: Exploratory Study
Source: J Med Internet Res. 2017 Oct 13;19(10):e352. doi: 10.2196/jmir.7656 (PMC5660293; doi:10.2196/jmir.7656)
Supplement: Multimedia Appendix 1 [file jmir_v19i10e352_app1.pdf]

Appendix 1. Focus group discussion guide

This project aims to investigate consumers' self-reported participation on social networking sites and other online forums for health purposes.

1. Tell me about your use of social media/ SNS for health, disease and medicines information.

   Prompts:
   - *a) Which one(s) have you used and which ones are you currently using?*
   - *b) When did you first start?*
   - *c) Are you an anonymous user or use a pseudonym/ nickname in your profile; or do you use your actual name?*
   - *d) What were/are your motivations to join/access?*
   - *e) If stopped using or changed are there any particular reasons that you stopped/ changed online forums/ SNSs?*
   - *f) Which technological devices do you use to access social media/SNS (e.g. computer, smartphone, tablet)*
2. How active would you say you are in the online forums/SNS we have just discussed?

   Prompts:
   - *a) How often and when is it used?*
   - *b) Do you observe, engage in discussions, ask questions, provide information, and/ or start discussions topics?*

Objective: Explore how consumers identify and evaluate SNS for health related purposes.

3. Referring back to the social media websites you mentioned, how did you discover them?
4. How did you select the online forum/SNS groups?

   Prompts:
   - *a) What criteria did you use to assess/ judge the group?*
   - *b) How do you evaluate that it is worth joining and continuing to participate?*

Objective: Investigate what kind of online health information consumers seek and request from others.

5. What kind of health, disease and medicine information have you sought from social media/ SNS?

   Prompts:
   - *a) What (were the factors that) made you trust that piece(s) of health, disease and medicine information? Any double-check mechanism?*
   - *b) How useful is it for you to see other people's health/ medication-related posts?*
   - *c) How convenient is social media in helping you to get the information you need?*
   - *d) How useful/effective is social media in helping you to get the information you need?*

6. Why did you get information from social media instead of anywhere else (e.g. institutions websites, health care setting/professionals)?

Objective: Explore the type of information consumers share with others publicly.

7. What kind of medicine information have you provided/ posted (personal clinical information, personal experience, latest scientific information)?

Prompts:

a) *What influences you to post information in an online forum?*
b) *Does the information you provide online differ depending on the SNS and any other circumstances? Feedbacks?*

8. How comfortable are you in providing health/ medicines information to other people?

Prompts:

Objective: Determine the impact of these online activities on their decision making behaviour;

9. How has your participation in these online venues changed or influenced your health decision-making behaviour (e.g. adherence, treatment changes)?

Prompts:

a) *Which topics were most influential?*
b) *Which topics were least influential?*

Objective: Determine the impact of these online activities on healthcare service usage/ perception.

10. How has participation on online forums / SNS groups changed the way you perceive healthcare professionals? Why/ reasons?
    - *Positively or negatively? Tell me the reasons*
    - *How you compare it with regular health services/ interaction?*

Objective: Explore if consumers are interacting with healthcare professionals on social media and if so, what kinds of interaction takes place.

11. How has participation on online forums / SNS groups changed the way you interact with the healthcare system/ healthcare professionals? Why/ reasons?

12. Have you had contact with healthcare professionals (or health services) through online forums/SNS?
    a) Which ones?
    b) Please describe the interactions / services received.

Objective: Determine the opinions about, and level of satisfaction with, their use of SM for health purposes.

13. Overall how do you perceive the online health environment and why?
    a) Advantages? Please explain.
    b) Do you have an issue with the use of SM for health related reasons? Explain
