# Supplementary material for: Consumer Health-Related Activities on Social Media: Exploratory Study
Source: J Med Internet Res. 2017 Oct 13;19(10):e352. doi: 10.2196/jmir.7656 (PMC5660293; doi:10.2196/jmir.7656)
Supplement: Multimedia Appendix 2 [file jmir_v19i10e352_app2.pdf]

Appendix 2. Participants' demographics.

Characteristic	Participants (n=36)
Sex	
Female	17 (47%)
Male	19 (53%)
Age (years)	
Range	27 – 71
Mean ± SD	47.3 ± 10.2
Country of birth	
Australia	26 (72%)
England	2 (5.5%)
New Zealand	2 (5.5%)
Other	6 (17%)
Education	
Less than High School	3 (8%)
High School	11 (30%)
College or Technical Education (TAFE)*	6 (17%)
Undergraduate	12 (33%)
Postgraduate	2 (6%)
Data missing	2 (6%)
Employment status	
Full-time	18 (50%)
Part-time	9 (25%)
Home duties	3 (8%)
Retired	2 (6%)
Unemployed	4 (11%)
Self-reported health status	
Excellent/very good	14 (39%)
Fair	17 (47%)
Poor/very poor	5 (14%)

* TAFE: Technical and Further Education
